# Quantitative and qualitative Data on historical Vertebrate Distributions in Bavaria 1845

**DOI:** 10.1038/s41597-025-04846-8

**Published:** 2025-03-28

**Authors:** Malte Rehbein, Belen Escobari, Sarah Fischer, Anton Güntsch, Bettina Haas, Giada Matheisen, Tobias Perschl, Alois Wieshuber, Thore Engel

**Affiliations:** 1 Chair of Computational Humanities, University of Passau, Passau, Germany; 2 Freie Universität Berlin, Botanic Garden and Botanical Museum Berlin. Center for Biodiversity Informatics and Collection Data Integration (ZBS), Berlin, Germany; 3 Research Institute for Farm Animal Biology (FBN) Dummerstorf, Dummerstorf, Germany; 4 Generaldirektion der Staatlichen Archive Bayerns, Munich, Germany; 5 Institute of Biodiversity, Friedrich Schiller University Jena, Jena, Germany; 6 Department of Biodiversity and People, Helmholtz Centre for Environmental Research - UFZ, Leipzig, Germany; 7 German Centre for Integrative Biodiversity Research (iDiv) Halle-Jena-Leipzig, Leipzig, Germany

**Keywords:** Biodiversity, Forestry, History, Interdisciplinary studies

## Abstract

Archival collections contain an underutilized wealth of biodiversity data, encapsulated in government files and other historical documents. In 1845, the Bavarian government conducted a comprehensive national survey on the occurrence of 44 selected vertebrate species across the country. The detailed expert responses from 119 forestry offices, totalling 520 handwritten pages, have been preserved in the Bavarian State Archives. In this study, we digitized, annotated, geographically referenced, and published these historical records, making them widely available as data for research and conservation planning. Our dataset, openly accessible through the Global Biodiversity Information Facility (GBIF) and Zenodo, contains 5,467 species occurrence records from 1845. Besides the binary presence/absence data, we have also published the original textual survey responses, which contain rich qualitative information, such as species abundances, population trends, habitats, forest management practices, and human-nature relationships. This information can be further processed and interpreted to address a range of questions in historical and contemporary ecology.

## Background & Summary

‘Die Wildkatze ist leider noch nicht gänzlich ausgerottet, und jedes Jahr liefert noch einige Exemplare.’ (Unfortunately, the wildcat has not yet been completely eradicated, and every year still yields a few specimens.) (Johann Mannert, head of the forestry office in Eltmann, Lower Franconia, 9 September 1845).

Natural history museums^1^ and archival collections play a crucial role in preserving historical species records for biodiversity research and conservation, and documenting biodiversity changes throughout the Anthropocene. While the past two decades have seen rapid growth in digitizing museum specimens^2^, other historical records, especially textual sources of biodiversity remain largely untapped. These sources, typically found in “archives of societies”^3^, include administrative files, correspondence, maps, audiovisual records, and other forms of grey literature and media^4^. Unlike “archives of nature”, such as fossils, pollen, or soil core samples, and contemporary species occurrence records, “archives of societies” require specialized expertise in historical sciences to be discovered, meaningfully interpreted, and effectively used for ecological research. This challenge has led to the emergence of historical ecology—an interdisciplinary field bridging the biological sciences with societies’ archival records^2,5^.

Some ecological studies have already utilized historical data such as those by Clavero *et al*.^6^ and by Nores and López-Bao^7^, both of whom used 19th-century geographical dictionaries to infer historical distributions of wolves in Spain, attempting to provide a baseline for current species conservation efforts, as did Clavero and Delibes for the lynx in Spain on a long time span^8^. Viana *et al*. harvested, analysed, and published data from a historical survey across 16th-century Spain^9^. Similarly, Govaerts drew on archaeological and historical sources of financial administration to document the occurrence of 46 bird species in late mediaeval Holland^10^. Another frequently used type of historical source is land and forest surveys, some of which date back to the 18th century. For example, Hanbery, Palik, and He compared an 1812 land survey with current forest surveys to examine shifts in forest community composition and biotic homogenisation in Minnesota, USA^11^. In Europe, Gschwantner *et al*. provide a review of historical forest inventories^12^. However, most literature on historical land and forest surveys focuses on tree communities and vegetation, with little reference to faunistic data. Furthermore, historical species records are rarely made accessible through biodiversity research infrastructures, such as the Global Biodiversity Information Facility (GBIF), except zoological and botanical collections. One possible reason for the lack of historical data is that, to be useful for biodiversity research, raw historical data must first be discovered, then understood, interpreted, annotated, and enriched in a process we refer to as “valorization”^13^.

**Fig. 1:**
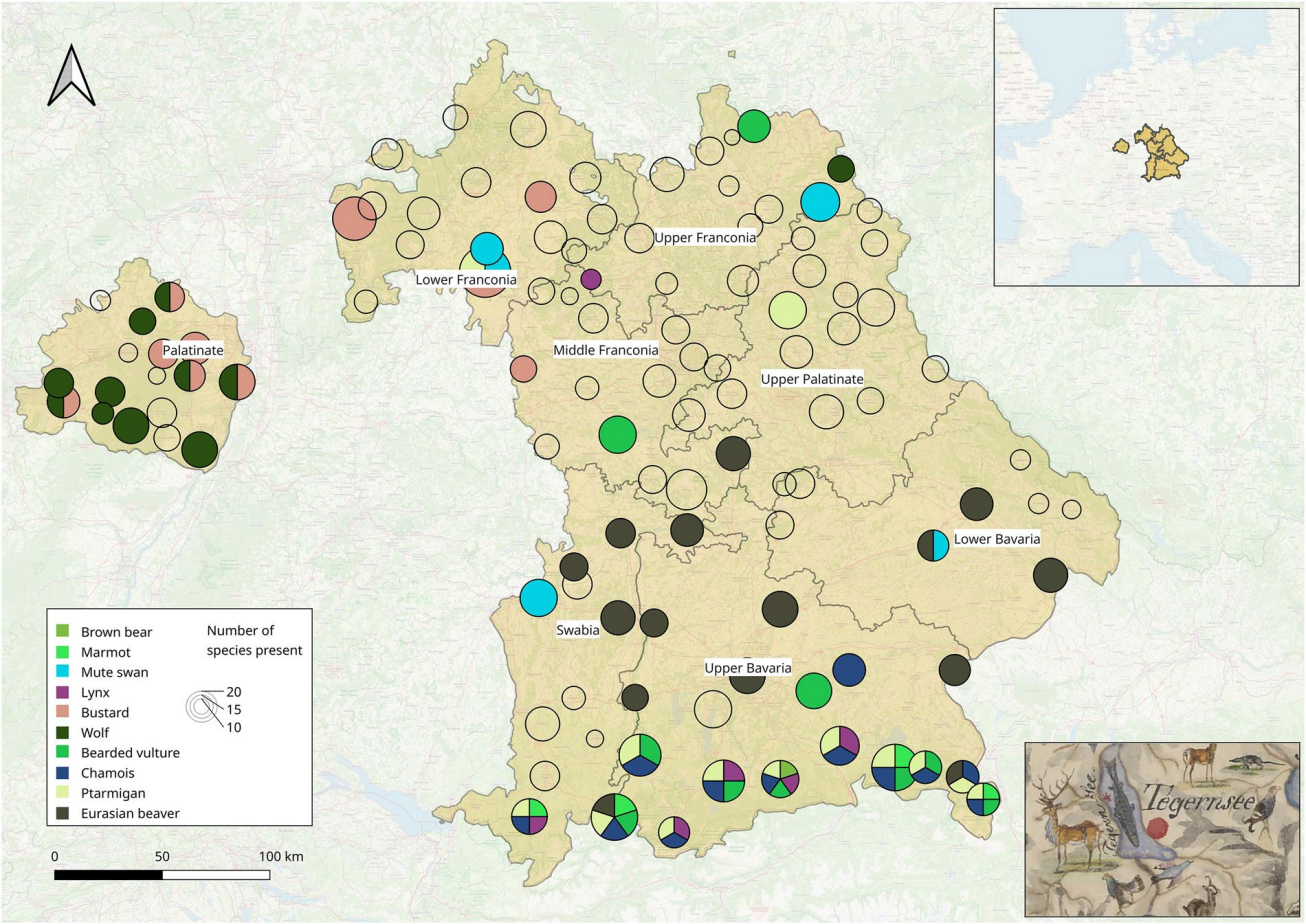
Forestry Offices and Rarest Species. The map displays the locations of all 119 forestry office seats as circles, with the circle diameters representing the number of species reported as present in each district. The ten rarest species in the survey are colour-coded by their presence. The map also includes the administrative districts of the Kingdom of Bavaria as they existed in 1845. The bottom right image shows a part of Wagner’s map from 1846. Map sources: own analysis; OpenStreetMap (OSM); QGIS; Bayerische Staatsbibliothek, BSB Cod.icon. 180 rc; HGIS Germany (https://hgl.harvard.edu/catalog/harvard-ghgis1848districts).

The dataset presented here was acquired from the Bavarian State Archives, resulting from interdisciplinary collaboration between natural sciences and humanities. We processed extensive historical sources on the occurrence status of 44 selected vertebrate species from a nation-wide standardized survey conducted in the Kingdom of Bavaria in 1845. The original source comprises the responses of 119 forestry offices, each contributing four handwritten pages. We transformed this traditional ecological knowledge—520 pages of handwritten text—into 5,467 classified, annotated, and geographically coded data points, now available on Zenodo^14^ and GBIF^15^ to support research in historical ecology (see Fig. 1 for an overview). In addition to binary species occurrence records (i.e. presence/absence), our dataset also includes transcribed qualitative responses from the forest officers who filled in the survey, as well as their names (see the opening quote for an example). These transcripts often provide valuable insights beyond simple presence or absence data.

The data are valuable for addressing a range of research questions in both contemporary and historical ecology, meeting the critical need for historical species occurrence records and long-term time series that help better understand biodiversity changes in the Anthropocene^16^. Historical species records, when critically assessed, can inform conservation baselines, national red lists, and policy-making^17^. The quote at the beginning of this article illustrates how qualitative data also offer insights into historical human-nature relationships. Additionally, these records provide information on changing species habitats, precise locations of animal sightings, and possible reasons for population declines and local extinctions. Being informed from the past^18^ is “vitally important”^19^ for various ecological applications, including restoration and conservation efforts, where historical baselines are essential^8^.

Aside from a report by the conductor of the survey and a brief mention in a biography^20^, the survey and its data have received little attention in the literature. Neither a systematic evaluation of the survey nor a publication of the original sources has been undertaken to date.

## Methods

### Study area

The data presented here encompass the entire Kingdom of Bavaria, then an independent nation; now, with some territorial changes, the largest federal state in Germany. This area includes a substantial territory (76,770 km^2^, of which 24,560 km^2^, or 31.99%, are woodland), almost twice the size of the Netherlands, and a variety of ecosystems, representing 21 of the 24 landscape types currently described by the German Federal Agency for Nature Conservation (https://www.bfn.de/daten-und-fakten/biogeografische-regionen-und-naturraeumliche-haupteinheiten-deutschlands). These include smaller parts of the Austrian Limestone Alps as well as left-bank Rhine regions, such as the Palatinate Forest adjacent to the Vosges Mountains along the French-German border. In 1845, the area was predominantly agricultural and pre-industrial with an overall population density of 57.8 inhabitants per km^2^ ^21^.

### Data processing (Overview)

**Fig. 2:**
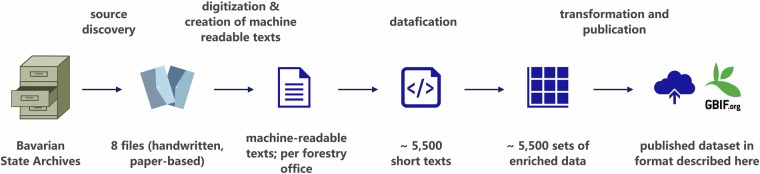
Data Processing Workflow (Overview). This diagram illustrates the workflow used to create the Historical Animal Observation Records by Bavarian Forestry Offices (1845) dataset, from the discovery of the archival sources to the publication of the final data in public repositories. The top row describes each processing step, while the bottom row outlines the resulting outputs.

Data processing (see Fig. 2) encompassed the following steps, which are described in more detail below:

- Source discovery and historical contextualization
- Digitization and creation of machine-readable texts
- Datafication
- Transformation and publication

**Fig. 3:**
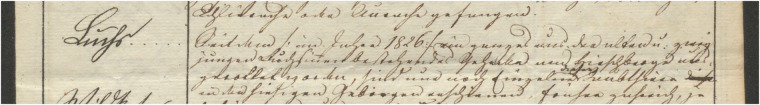
Sample Response. This image is part of the Tegernsee (Salforste) report (cf. Figure 4 for the full-page image), documenting the presence of the lynx. The transcribed original language, along with its English translation, is provided in the text. Source: BayHStA Zool. Staatssammlung, 217, p. 14.

Figure 3 shows an entry of a sample record in its original form from the Tegernsee (Salforste) office, describing the occurrence of the Eurasian lynx (*Lynx lynx*). The text is presented in both the original language and its English translation:

Luchs: “Seitdem /: im Jahre 1826:/ ein ganzes aus den alten u. zwey jungen Luchsinnen bestehendes Gehecke am Hirschberge ausgerottet worden, sind nur noch Einzelne dieser Raubthiere diese in den hiesigen Gebirgen erschienen, früher zahlreich, jedoch immer nur periodisch”

Lynx: “Since /: in 1826:/ an entire enclosure consisting of the old and two young female lynxes on the Hirschberg was eradicated, only individuals of these predators have appeared in the local mountains, formerly numerous, but always only periodically”

### Source discovery and historical contextualization

This study is based on historical documents from 1845 containing animal observations recorded by Bavarian forestry offices. Foresters used predefined, standardized forms sent to them to document the presence or absence of 44 listed animal species within their districts, as well as fish species in local waters. In some cases, foresters provided additional information on species beyond those listed on the forms. This source is particularly valuable due to its comprehensive coverage of all of Bavaria at a specific point in time—a rarity among archival sources. Observations were documented by a total of 119 forestry offices across the eight Bavarian government districts. The documents are organized into ten files, with one file for each of the eight government districts (Upper Bavaria, Lower Bavaria, Upper Franconia, Middle Franconia, Lower Franconia, Upper Palatinate, Palatinate, Swabia-Neuburg), a file for the Salforste region—a forested area located in the border region between Germany and Austria, historically managed as a shared resource between Bavaria and Austria—and a summary for all of Bavaria. Together, these documents comprise approximately 520 pages.

The documents are housed in the Bavarian State Archives as part of the record group for the Zoological State Collection (https://www.gda.bayern.de/service/findmitteldatenbank/Kapitel/0ea38d12-d425-4b3e-a497-7b6830f439e1). This record group, spanning the period from 1827 to 1990, consists of 1,442 archival units. These units include documents related to the organisation, administration, and personnel matters of the Zoological State Collection, along with records of acquisitions, inventory catalogues, professional staff correspondence, and scientific research. The animal occurrence records completed by the forestry offices were originally held in the Zoological State Collection in Munich and were transferred to the Bavarian State Archives in 2013.

The context of these documents is deeply intertwined with the complex history of the Zoological State Collection. Established in 1827 as an independent public research institution by order of King Ludwig I, the Zoological Collection was initially separated from the scientific collections of the Bavarian Academy of Sciences. However, it soon became dependent on Ludwig Maximilian University, which had to transfer its zoological collection to the state cabinet. The collection’s director, or conservator, was typically the university’s Professor of Zoology. Simultaneously, the “General Conservatory of State Scientific Collections” was founded, intended to secure the collections’ independence. However, this agency’s effectiveness was limited by the Academy’s influence, as its president also served as the General Conservator. As a result, the zoological cabinet existed in an ambiguous organizational and personnel structure, with its scientific staff primarily sourced from the university, but required approval from the state collection administration, which was heavily reliant on the Academy’s resources.

The valuable dataset presented here originated from a commission by Bavarian Crown Prince Maximilian II, who tasked the zoologist Johann Andreas Wagner with mapping the distribution of Bavaria’s most significant animal species^22^. Wagner, who had been Professor of Zoology at the University of Munich and Deputy Curator of the State Zoological Collection since 1836, worked diligently to expand the collection and made notable contributions to zoological taxonomy^20^. To fulfil the prestigious royal commission and ensure a comprehensive survey of Bavaria’s animal population, Wagner decided to involve the royal forestry offices^22^. These offices were likely selected because the foresters were not only expected to possess a thorough knowledge of local wildlife but were also regarded as qualified experts due to their academic training.

**Fig. 4:**
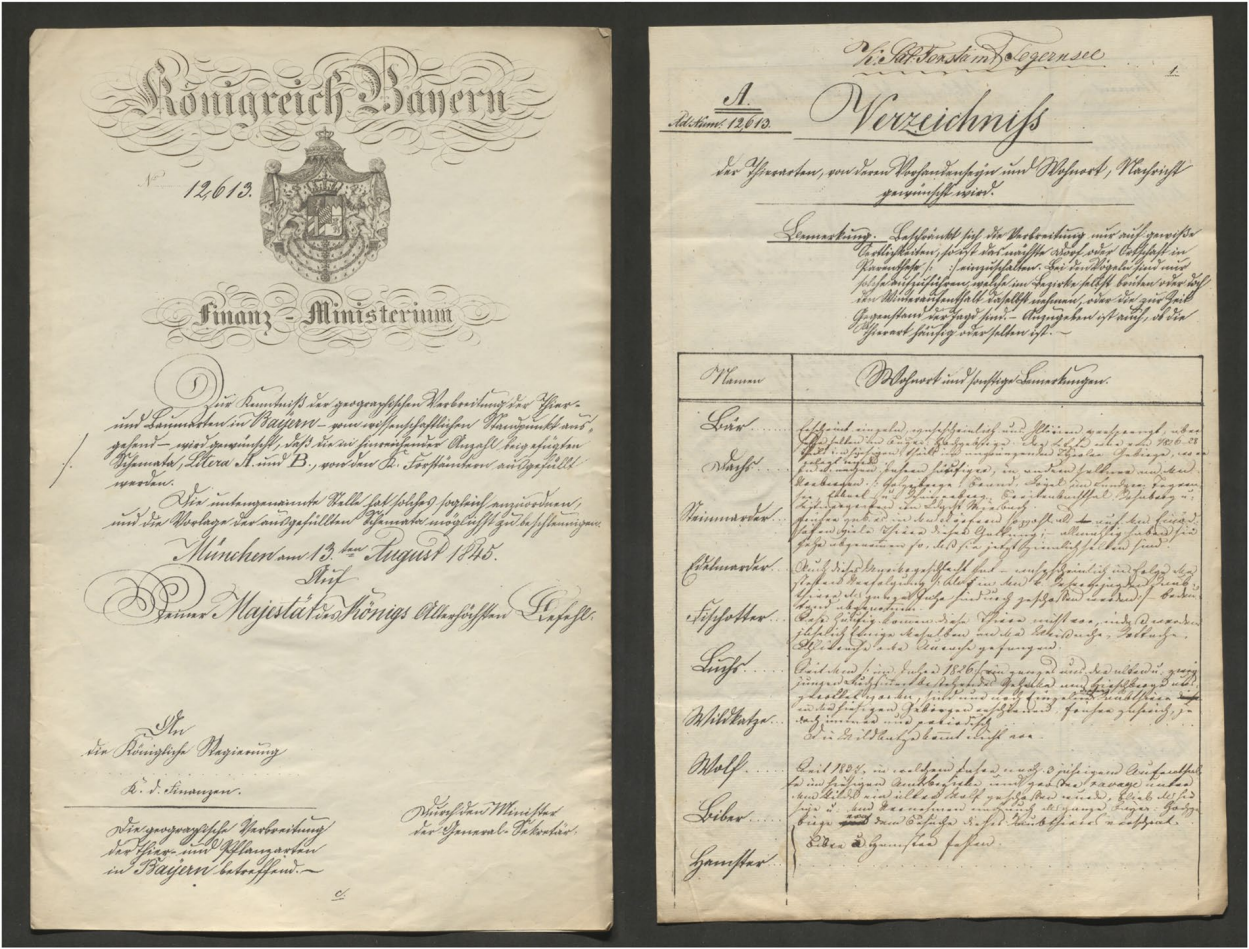
Royal Order and Tegernsee Response. Left: Royal order from 13 August 1845: “Zur Kenntniß der geographischen Verbreitung der Thier- und Baumarten in Bayern — vom wissenschaftlichen Standpunkt aus gesehen — wird gewünscht, daß die in hinreichender Anzahl beigefügten Schemata, Litera A. und B., von den K. Forstämtern ausgefüllt werden.” (For an understanding of the geographical distribution of animal and tree species in Bavaria—from a scientific perspective—it is desired that the attached forms, Litera A and B, be completed by the Royal Forestry Offices.) Source: BayHStA, Zoologische Staatssammlung, 208. Right: Completed first page of the survey form from the Salinenforstamt Tegernsee. Source: BayHStA Zoologische Staatssammlung, 217.

In 1845, the 119 royal forestry offices, then subordinate to the Bavarian Ministry of Finance, were issued a directive from the Ministry, mandating that they systematically record the animal species present in their respective forestry districts. The motivation behind the survey is evident from the official letter: foresters were explicitly asked to document the animals from a “scientific perspective” (cf. Figure 4). This initiative arose during a period of rapid scientific and economic development. Concurrently, there was a growing awareness of the relationship between humans and nature, with particular interest in the systematic and descriptive documentation of the natural world. While the geographical distribution of plants had already been well studied, Wagner observed that zoology still lagged behind in this regard^23^.

The survey responses, comprising more than 5,400 entries in short prose, offer insights not only into species occurrence but also into habitats, their changes, and the effects of human activity on wildlife populations. For example, the survey contains the last recorded sightings of the beaver (*Castor fiber*) in Bavaria before its extinction around 1867^24^. Wagner anticipated the beaver’s impending extinction, attributing it to particularly lucrative but officially prohibited hunting^22^.

The survey results formed the basis for a map visualizing animal distribution in Bavaria, as commissioned by the Crown Prince^25^. Andreas Wagner also presented these findings in a lecture at the Bavarian Academy of Sciences on 21 February 1846, where he had been a member since 1835. This lecture was subsequently published in *Gelehrte Anzeigen*, a journal issued by members of the Bavarian Academy of Sciences^22^.

### Digitization and creation of machine-readable texts

It is typical for many historical sources found in archives of societies that the raw data of the 1845 survey consist of paper-based archival files in which handwritten texts represent the information of interest. To enable further processing, we created digital images of these original documents following the German Research Council (DFG) recommendations^26^, specifically uncompressed TIFF images with a resolution of 300 ppi (pixels per inch) as master files to allow future reuse and upcycling^27^. We then transformed these image-based raw data into machine-processable texts through a two-step process: first, layout recognition (identifying regions and script baselines), and, second, content transcription using AI-based Handwritten Text Recognition (HTR) via the pre-trained Transkribus model “The Text Titan”, without additional custom training^28^. This automated process produced an initial Character Error Rate (CER) of approximately 7%, which was significantly improved through manual post-correction by trained palaeographers. The texts were transcribed “as is”—preserving the original language, maintaining spelling errors, and leaving abbreviations unresolved—to keep the transcriptions as faithful to the originals as possible.

### Datafication

These raw texts were organized by forestry office name and taxon name, creating a total of 5,467 entries for 44 predefined species, along with additional species reported by foresters. We interpret this processing of a non-digital artefact into computable codes as *datafication*^27^, whereby data of interest were extracted from textual content that was originally unstructured and not formatted for direct use. Instead, the information followed a free-text format, adhering to the instructions provided in Wagner’s questionnaire (presented here in English translation):

“List of animal species of whose existence and place of residence information is desired. Note: If the distribution is limited to certain localities only, the nearest village or locality should be included in parenthesis. In the case of birds, only those which breed in the district itself or which spend the winter there, or which are currently the subject of hunting, should be listed. – It should also be stated whether the species is common or rare.”

We applied the following rules for datafication:

1. **Metadata (per district):** For each of the 119 forestry districts, the location of its office seat was researched and geographically coded. *Example: “Forstverwaltung Deggendorf” → office seat Deggendorf, Lower Bavaria → longitude = 12.9603, latitude = 48.8348. Sources: survey headers, State Directory Kingdom of Bavaria 1845*^29^*, and OpenStreetMap (OSM)*.
2. **Species names:** Mapping of the predefined 44 species names and an additional 91 individually reported, partly historical, names to their scientific names and taxonomy (see Tables 1, 2). The trivial names and Latin names used in the historical dataset serve as the central point of integration for the data into the service infrastructures of biodiversity informatics, e.g., for the analysis of historical species distributions or changes in biodiversity patterns over time. However, for the interoperability of the data, it is necessary to understand the species concepts used and to link them to recent concepts using stable persistent identifiers. Beyond clarifying the concepts, this also avoids the problem of orthographic variants of species names during data integration. We used the checklist infrastructure of the German Federal Agency for Nature Conservation (BfN) for the taxonomic annotation of the data (https://checklisten.rotelistezentrum.de/api/public/swagger-ui). On the one hand, it provides the taxonomic and geographical coverage necessary for the annotation of forestry data. On a second level, the infrastructure allows taxonomic concepts to be precisely referenced using persistent identifiers, providing the basis for the semantic annotation of the taxon names used^30^. *Example: “Halbente” → Knäkente → *anas querquedula. *Sources: survey, checklist (e.g*., https://checklisten.rotelistezentrum.de/api/public/1/taxon/24804*); historical literature and dictionary in rare cases (e.g*., https://www.dwds.de/wb/dwb/halbente*)*.

**Table 1:** Species Overview (mammals).

Scientific species name	Species German name in source	Common species name in English	Earliest German record	German red list status (2024)	Number of existing German records in GBIF before 1850	Number of new records in source	Qualitative textual information (in tokens)
*Ursus arctos* Linnaeus, 1758	Bär	Brown bear	1901	Extinct or Lost	0	1	404
*Meles meles* (Linnaeus, 1758)	Dachs	Badger	1882	Not Threatened	0	116	1,397
*Martes foina* (Erxleben, 1777)	Steinmarder	Stone marten	1844	Not Threatened	1	116	459
*Martes martes* (Linnaeus, 1758)	Edelmarder	Pine marten	1870	Near Threatened	0	117	1,102
*Lutra lutra* (Linnaeus, 1758)	Fischotter	Eurasian otter	1838	Threatened	2	116	1,471
*Lynx lynx* (Linnaeus, 1758)	Luchs	Lynx	1959	Threatened with Extinction	0	7	416
*Felis silvestris silvestris* Schreber, 1777	Wildkatze	Wildcat	1834	Threatened	3	65	706
*Canis lupus* Linnaeus, 1758	Wolf	Wolf	1827	Threatened	2	11	507
*Castor fiber* Linnaeus, 1758	Biber	Eurasian beaver	1823	Near Threatened	2	16	541
*Cricetus cricetus* (Linnaeus, 1758)	Hamster	Hamster	1846	Threatened with Extinction	1	27	452
*Marmota marmota* (Linnaeus, 1758)	Murmelthier	Marmot	1934	Not Threatened	0	3	331
*Sus scrofa* Linnaeus, 1758	Wildschwein	Wild boar	1842	Not Threatened	2	22	710
*Cervus elaphus* Linnaeus, 1758	Edelhirsch	Red deer	1841	Not Threatened	2	88	1,394
*Dama dama* (Linnaeus, 1758)	Damhirsch	Fallow deer	1900	Not Evaluated	0	29	635
*Capreolus capreolus* (Linnaeus, 1758)	Reh	Roe deer	1851	Not Threatened	0	116	1,340
*Rupicapra rupicapra* (Linnaeus, 1758)	Gemse	Chamois	1871	Near Threatened	0	12	413

Overview of species recorded in the historical survey of 1845. The table lists all mammals of the survey, including their original, common English, and modern scientific names. It refers also to the German red list status as of October 2024 and already existing records in the GBIF database (mostly preserved specimen or material citations, but not human observations). Additionally, the size of qualitative information about the species is given in total number of tokens. Sources: aggregated data from the dataset, GBIF database, and the German Red List as of October 2024 (https://www.rote-liste-zentrum.de/).

**Table 2:** Species Overview (birds and reptiles).

Scientific species name	Species German name in source	Common species name in English	Earliest German record	German red list status (2024)	Number of existing German records in GBIF before 1850	Number of new records in source	Qualitative textual information (in tokens)
*Gypaetus barbatus* (Linnaeus, 1758)	Lämmergeier	Bearded Vulture	1988	Not Evaluated	0	12	365
*Aquila chrysaetos* (Linnaeus, 1758)	Steinadler	Golden eagle	1890	Extremely Rare	0	23	459
*Haliaeetus albicilla* (Linnaeus, 1758)	Seeadler	White-tailed eagle	1871	Not Threatened	0	17	385
*Pandion haliaetus* (Linnaeus, 1758)	Fischaar	Osprey	1828	Threatened	4	45	573
*Bubo bubo* (Linnaeus, 1758)	Uhu	Eurasian eagle-owl	1808	Not Threatened	7	57	713
*Corvus frugilegus* Linnaeus, 1758	Saatkrähe	Rook	1816	Not Threatened	32	93	787
*Pyrrhocorax graculus* (Linnaeus, 1766)	Alpendohle	Alpine (yellow-billed) chough	1894	Extremely Rare	0	26	417
*Pyrrhocorax graculus* (Linnaeus, 1766)	Steinkrähe /: corvus graculus:/	Unclear, possibly: red-billed chough	—	—	—	22	384
*Luscinia megarhynchos* C.L. Brehm, 1831	Nachtigall	Nightingale	1812	Not Threatened	23	73	875
*Tichodroma muraria* (Linnaeus, 1766)	Mauerspecht	Wallcreeper	1917	Extremely Rare	0	42	396
*Tetrao urogallus* Linnaeus, 1758	Auerhuhn	Western capercaillie	1816	Threatened with Extinction	27	74	863
*Tetrao tetrix* Linnaeus, 1758	Birkhuhn	Black grouse	1860	Threatened with Extinction	0	82	992
*Tetrastes bonasia* (Linnaeus, 1758)	Haselhuhn	Hazel grouse	1823	Highly Threatened	15	58	695
*Lagopus muta* (Montin, 1781)	Schneehuhn	Ptarmigan	1855	Extremely Rare	0	14	370
*Phasianus colchicus* Linnaeus, 1758	Fasan	Pheasant	1818	Not Evaluated	10	23	490
*Otis tarda* Linnaeus, 1758	Trappe	Bustard	1810	Threatened with Extinction	7	10	313
*Scolopax rusticola* Linnaeus, 1758	Schnepfe	Sandpiper	1815	Near Threatened	19	118	1,410
*Gallinago gallinago* (Linnaeus, 1758)	Bekassine	Common snipe	1815	Threatened with Extinction	48	104	1,132
*Grus grus* (Linnaeus, 1758)	Kranich	Common crane	1836	Not Threatened	1	30	440
*Botaurus stellaris* (Linnaeus, 1758)	Rohrdommel	Eurasian bittern	1810	Threatened	9	50	635
*Ciconia ciconia* (Linnaeus, 1758)	Weißer Storch	White stork	1817	Threatened	12	73	873
*Ciconia nigra* (Linnaeus, 1758)	Schwarzer Storch	Black stork	1841	Not Threatened	1	20	481
*Cygnus olor* (J.F. Gmelin, 1789)	Höckerschwan	Mute swan	1887	Not Threatened	0	5	403
*Cygnus cygnus* (Linnaeus, 1758)	Singschwan	Whooper swan	1838	Extremely Rare	2	17	498
*Anser anser* (Linnaeus, 1758)	Gemeine Wildgans	Common wild goose	1844	Not Threatened	1	94	1,141
*Anser fabalis* (Latham, 1787)	Saatgans	Bean goose	1840	Not Evaluated	1	64	751
Aves Linnaeus 1758	Enten	Ducks	n/a	n/a	n/a	n/a	n/a
*Vipera berus* (Linnaeus, 1758)	Kupferotter	Common European adder	1834	Highly Threatened	4	60	775

Overview of species recorded in the historical survey of 1845. The table lists all birds and reptiles of the survey, including their original, common English, and modern scientific names. It refers also to the German red list status as of October 2024 and already existing records in the GBIF database (mostly preserved specimen or material citations, but not human observations). Additionally, the size of qualitative information about the species is given in total number of tokens. Sources: aggregated data from the dataset, GBIF database, and the German Red List as of October 2024 (https://www.rote-liste-zentrum.de/).
3. **Text:** Text is transcribed character by character as is in the original source including abbreviations and potential misspelling. Unsure transcriptions are marked as ‘#…#’. A significant amount of entries repeat the previous line by using ‘ditto’ words or signs, or they aggregate entries by using curly brackets. Occurrence data have been drawn from the line referred to by the signs or brackets in these cases. *Example: “Lynx: Kömmt nicht vor; Wild cat: dito; Wolf: dito” yields occurrence = 0 for all three species (Deggendorf, Lower Bavaria)*.
4. **Occurrences:** Binary classification (absence or presence) of occurrences per species and district. This basic classification of occurrence also caters for the fact that the responses by the forestry offices were quite diverse in scope and quality. On the lower end of the scale of detail, an entry could simply read “[species] does not exist here” or even only a strike-through of the species names (both classified as ‘0’) while on the upper end, detailed information about habitat or specific location could have been given. In cases of reference to additional species or more comprehensive reporting such as specific time of observation, a new entry has been created. *Source: survey*.
  - **Classification as 1:** We could draw from the texts that the reporting office was sure or at least provided good reason that a specimen of this species had recently (in 1845) been seen within the boundaries of its district. We did not distinguish the type of the animal’s visit (e.g. resident or migratory bird); nor have statements about quantity and abundance (yet) been datafied (e.g. rare occurrence, close to extinction, or regular). Where such information is available, future research may extract it from the texts given in the dataset. *Example: “The hamster is found only very rarely.” (Freysing, Lower Bavaria)*.
  - **Classification as 0:** There is no or no justifiable evidence for an observation. *Example: “The bear is not present.” (Freysing, Lower Bavaria)*. This also covers the few cases in which an occurrence is reported to be situated outside a natural environment (in game parks or pheasantries). *Example: “The pheasant can only be found in pheasantries” (Bayreuth, Oberfranken)*.
5. **Time:** We generally assume all reports refer to the year 1845 or only few years earlier if not otherwise stated. Where otherwise stated, the given date has been coded and used as time of observation. In case of the latter, a new data entry has been created (see above). Examples: “has not occurred since 1710, when the last one was seen in the former Rehau district and caught in the Sparneck district” — > creates two entries, one for 1710 (classified ‘1’), one for 1845 (classified ‘0’). Source: survey.
6. **Location:** We generally assume all occurrences are located within a default radius of 20 km from the seat of the forestry office.

### Transformation and publication

Finally, all data were transformed using a standardized terminology based on the Access to Biological Collection Data (ABCD) Schema (http://www.tdwg.org/standards/115). This transformation can also be seen as “valorisation”^13^—creating value and impact from the historical sources, the knowledge they contain, and the historian’s expertise required for their mobilisation. We have published the data under a CC BY licence in two formats

1. A dataset uploaded to the *Global Biodiversity Information Facility* (GBIF) and published via the GBIF data services and the GBIF-hosted portal *Lebendiger Atlas der Natur Deutschlands* (LAND, https://land.gbif.de). Here, the data can be used within the given framework and its retrieval and visualization tools, and in the broader contexts of the entire GBIF database, allowing for spatial and temporal analysis as well as species comparisons^15^.
2. A dataset uploaded to the online repository Zenodo, available for download for in-depth analysis using statistical and text mining tools and Geographic Information Systems (GIS). This format also supports cross-disciplinary applications^14^.

## Data Records

The following data records are available:

1. Digital images from the original archival sources (https://www.gda.bayern.de/service/findmitteldatenbank/Kapitel/0ea38d12-d425-4b3e-a497-7b6830f439e1);
2. A dataset available at Zenodo, containing^14^:
  Transcription data (as XML file);
  Main occurrence data table (as CSV file);
  Species names data table (as CSV file);
  Forestry offices and administrative districts data tables (as CSV file);
  Additional information data table (as CSV file).

High-resolution digital images of all original source files from the digitization process are accessible via the online service of the Bavarian State Archives, organized in Bavarian administrative districts and including the file for the Salforste region. The images are displayed in the original order of the files, with full navigation and image manipulation options available through the DFG Viewer (https://dfg-viewer.de/). Users can also utilise the Open Archives Initiative Protocol for Metadata Harvesting (OAI-PMH) interface for downloading or linking metadata. To enable verification of the transcriptions, the main data table includes a direct URL linking to the specific digital image corresponding to each entry.

The data repository includes the transcription results in both human-readable and machine-readable XML formats. Metadata in this file align with the archival metadata used by the Bavarian State Archives in their catalogues and the online archive. Metadata fields follow the standard of the Text Encoding Initiative (TEI, https://tei-c.org). The file content maintains the original order and is hierarchically organized as follows: administrative district → forestry office → species record. Text fields present entries “as is,” with no interpretation, correction, annotation, or analysis. These transcriptions allow users to verify each entry in the main data table without requiring palaeographic expertise.

The main occurrences data table contains:

- 5,467 records of observation data in structured CSV format, following the hierarchical structure of administrative district → forestry office → species with one row per species and forestry office;
- Additional rows for special cases: reported extra species, earlier (than 1845) observations reported;
- Transcriptions of the textual evidence for each entry (see Table 3 for a content breakdown);

**Table 3:** Qualitative breakdown of textual entries.

Entries containing…	Total number (of which are positive, i.e. presence entries)	Percentage of positive entries (N = 2,514)
habitat descriptions	832 (812)	32.3%
attributable toponyms	788 (782)	31.3%
datable references to occurrences before 1845	28	n/a
categorial quantifiers beyond binary presence/absence (such as häufig/often, selten/seldom)	1,708	64.9%

Estimates along four categories of how many of the text entries contain qualitative information beyond the pure core question of presence/absence of the species.
- Direct links to the digital images carrying the textual evidence;
- Identifiers linking each entry to the respective species and forestry office tables.

The species names data table contains:

- Species names in historical writing, English translation, and modern scientific names;
- Direct references to the German Red List checklist dataset.

The forestry offices and administrative districts data tables contain:

- Names and location (longitude and latitude) of the seats of each of the 119 forestry offices to allow approximate geographical coding of each observation;
- Names and English transcriptions of the eight administrative districts of the Kingdom of Bavaria in 1845.

Additional information about the forestry offices and officers, sourced from the 1845 Bavarian State Directory, is provided in a separate table^29^. For peer review, version 1.3 of the dataset has been used (https://zenodo.org/records/14008158).

## Technical Validation

The data presented here are derived from text-based historical sources, which means that the resulting dataset represent the original observations indirectly in two respects^31^: first, we did not observe the animals ourselves; and second, we did not directly question the foresters who reported these observations. Instead, as is typical and necessary for historical data drawn from societies’ archives, we critically relied on the traces^32^ left behind by foresters in their written reports from 1845. This approach raises methodological questions and challenges, closely tied to questions of data validation^33^. As with any work in historical ecology, an interdisciplinary approach was necessary and was applied to ensure reliable and verifiable data.

To validate the historical information found in the original documents, we employed the widely accepted methods of historical source criticism. Overall, there is no doubt that these sources are authentic and accurately represent what they claim to be.

We vetted the following:

1. **Origin of responses:** The survey documents were exchanged via the official postal service, some responses were hand-signed. The names of the signatories match those in the official register of forest managers in the 1845 State Directory of the Kingdom of Bavaria^29^.
2. **Integrity and completeness of the archival record:** The authorities have well-documented the provenance of these files, and the sources show no indication of later manipulations. A response exists for each forestry office listed in the State Directory^29^, suggesting that no entries are missing.
3. **Dating:** There is no doubt that the survey took place in 1845. The ministry’s executive order dates to that year, the names of forestry officers in the responses align with those in the State Directory issued annually^29^, and Wagner’s 1846 report to the Academy confirms the survey’s timing.
4. **Trustworthiness of content – intentional errors:** While individuals might have had motives to misrepresent or conceal information (e.g., involvement in wildlife poaching), it is highly unlikely for forestry officers, who were royal officials, to act deceptively. Given their duty to uphold official responsibilities without personal gain, intentional misreporting is improbable.
5. **Trustworthiness of content – unintentional errors:** Forestry officers possessed sound knowledge of fauna and flora, as they were required by law to undergo three years of specific training, including zoology, at a renowned university-level institution in Aschaffenburg or Munich^34^. We verified this for individual officials using their personnel files in the State Archives.
6. **Variability in response quality**: As noted, there are significant differences in the quality and scope of the responses, suggesting that foresters provided answers with varying levels of commitment. This variability imposes limitations on the comparability of responses, particularly in cases involving sightings of rare animal species.

Secondly, we validated data processing through the following methods:

1. Transcriptions were double-checked, and in cases of uncertainty, triple-checked by independent reviewers. Names of individuals, forest districts, and sub-districts were verified against the 1845 State Directory^29^, while place names were cross-referenced using historical gazetteers, regional websites, and historical maps. Unclear readings were marked accordingly. All transcriptions can be validated against the digitized originals, with references provided in the dataset.
2. Binary occurrence classifications were independently double-checked. These classifications can be verified at any time using the texts within the dataset.
3. Species names and classifications were verified, with rare, uncertain cases discussed and resolved based on the likelihood of species occurrence in Central Europe.
4. Consistency and completeness checks were automatically performed by script for each dataset version. This script validated the internal logic of entities and relationships by:

- Ensuring that each entry in *AOD_Observation_Record_Data.csv* has matching values for *OfficeName* and *District* in *AOD_Offices.csv*;
- Ensuring that each *SpeciesName* in *AOD_Observation_Record_Data.csv* corresponds to a valid entry in *AOD_Species_Names.csv;*
- Checking that each *Date* in *AOD_Observation_Record_Data.csv* falls within a reasonable range (1600–1845);
- Validating *AOD_transcriptions.xml* against its schema and ensuring that each core observation entry in *AOD_Observation_Record_Data.csv* has a corresponding XML element in *AOD_transcriptions.xml*.

## Usage Notes

The data presented here can be regarded as scientifically influenced traditional ecological knowledge^35^, with studies using this data classified as applied historical ecology—that is, “the use of traditional knowledge (TK) to help address problems of people and the environment today”^36^. As archives of societies likely hold extensive additional knowledge, such as records from past foresters—our approach to mobilising these sources may serve as a model for similar projects worldwide.

When using this dataset for research, it is essential to consider typical characteristics of historical data. Here are specific points to keep in mind:

### Geographical information

- **Changing Boundaries and Land Use:** Just as land use, habitats, and ecosystems have changed over time, so too have administrative boundaries. Therefore, geographic analysis requires using maps and shapefiles that accurately reflect the relevant period, such as those provided by GeoPortal Bayern (https://geoportal.bayern.de/geoportalbayern/suche/suche?0&q=historisch) or HGIS Germany (https://hgl.harvard.edu/catalog/harvard-ghgis1848districts).
- **Geographic Resolution of Observations:** The geographic resolution in this dataset is based on the administrative seats of forestry offices, giving each observation an average coverage area of 645 km^2^. The Salforste region is a special case: due to a bilateral treaty between Bavaria and Austria (the *Salinenkonvention* of 1829 which remains in force today), the jurisdiction—and thus the observations—of Bavarian forestry offices extend into parts of Austria^37^.
- **Detailed Location Information:** Some observations contain more specific location details. To make use of this higher geographic resolution at the data-entry level, geographic coding of the qualitative textual information is required, which is best supported by historical gazetteers and maps Local knowledge may also be helpful in certain cases.

### Time considerations

- **Observation Timing:** “1845” does not necessarily indicate a precise observation date; rather, it reflects a forester’s assessment in summer 1845 that the species in question was generally present (or absent) within his jurisdiction.

### Species and taxonomy

**Fig. 5:**
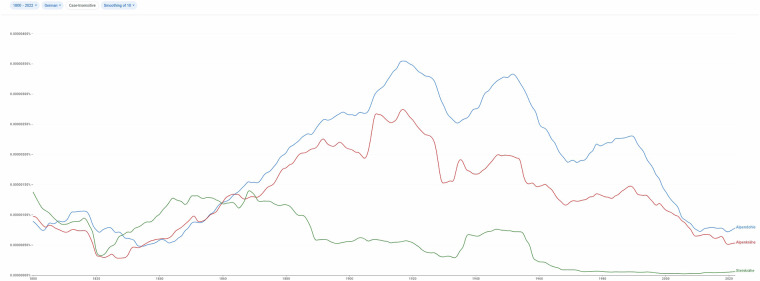
Usage of Species Names (1800–2022). Historical usage trends for the terms “Steinkrähe” (blue), “Alpenkrähe” (red), and “Alpendohle” (green) show that all three terms were used relatively evenly (though not synonymously) around 1845. Since then, “Steinkrähe” has become uncommon, leaving only “Alpendohle” (yellow-billed) and “Alpenkrähe” (red-billed) in common use. Source: Google Books Ngram Viewer (https://books.google.com/ngrams/).

- **Classification Limitations:** While we have classified the observation records to the best of our knowledge, there may occasionally have been misunderstandings by foresters about the species being surveyed. In such cases, interpreting the original text may be necessary, especially since foresters often explained their interpretations of the questionnaire.
- **Additional Species Mentions:** Mentions of additional species by foresters are uneven and unsystematic. Unlike the primary 44 species, an absence of reporting on these species does not imply their absence; rather, it simply means no information was provided.
- **Ducks Family Reporting:** Ducks were surveyed as a family, resulting in varied responses, from general statements (“ducks occur”) to detailed species lists. For general mentions, absence of notes on specific species (e.g., *Anas platyrhynchos*, mallard) does not imply the species did not occur, but rather reflects a lack of specific information.
- **Special Cases** – SP_0023 (“Alpendohle”) and SP_0024 (“Steinkrähe /: corvus graculus:/”): Differentiation between these two terms may be inconsistent in the data, as some foresters appeared uncertain. “Steinkrähe,” clarified by Wagner as “*corvus graculus*,” is no longer common in German (cf. Figure 5) and may historically refer to the red-billed chough (*Pyrrhocorax pyrrhocorax*)^38^. To approximate accurately, consider the red-billed chough (“Alpenkrähe” in modern German, https://www.gbif.org/species/2482552) and yellow-billed chough (“Alpendohle”, https://www.gbif.org/species/2482553), though differentiation within the responses may not be consistent.

### Qualitative information

- **Historical Language Considerations:** Keep in mind that language, and word meanings in particular, change over time. For example, the German term “merkwürdig” meant “notable” in the 19th century, but now means “bizarre.”
- **Subjectivity and Context:** The textual data provide subjective statements that need historical contextualisation and interpretation, particularly regarding baseline conditions^39^. Terms like “häufig” (frequent, often) may have different implications among respondents and may have had shifted in meaning and their baselines over time^40^.

The dataset opens up research questions beyond statistical analysis, including:

- **Influences on Species and Ecosystems:** Foresters reported not only species occurrences but also—though inconsistently—on factors affecting species’ living conditions, such as human actions, landscape changes, and climate influences^41^. Since the survey took place at the end of the European Little Ice Age, this contextual information could be particularly relevant. The dataset also provides insights into human attitudes towards animals—such as hunting—along with detailed habitat and location data. This content offers potential for studies in geographic anthropology and historical biology. To support such research, further semantic enrichment and annotation of the data would be beneficial.
- **Comparative and Combined Analyses:** The dataset can be compared or integrated with similar datasets from other regions or periods. Additionally, further research is needed to develop automated methods for data mobilisation and analysis of archival records^42^.
- **Advancing Biodiversity Informatics:** This work contributes to mobilising historical data that can be used to analyze ecosystem patterns and species distributions, as well as to explore underlying causes. Semantic annotation of species names with stable, persistent identifiers is an essential step towards integrating this dataset into a global biodiversity knowledge graph^43^. To enhance integration within the interconnected field of biodiversity informatics, further annotation of data on individuals, habitat types or other critical concepts could provide a foundation for improved automated data integration and inference^44,45^.

## Data Availability

No custom code has been used.
